# Electrochemical Behavior of Plasma-Nitrided Austenitic Stainless Steel in Chloride Solutions

**DOI:** 10.3390/ma17174189

**Published:** 2024-08-24

**Authors:** Viera Zatkalíková, Petra Drímalová, Katarzyna Balin, Martin Slezák, Lenka Markovičová

**Affiliations:** 1 Department of Materials Engineering, Faculty of Mechanical Engineering, University of Žilina, Univerzitná 8215/1, 010 26 Žilina, Slovakia; petra.drimalova@fstroj.uniza.sk (P.D.); martin.slezak@fstroj.uniza.sk (M.S.); lenka.markovicova@fstroj.uniza.sk (L.M.); 2 A. Chełkowski Institute of Physics, University of Silesia in Katowice, 75 Pułku Piechoty 1, 41-500 Chorzów, Poland; katarzyna.balin@us.edu.pl

**Keywords:** austenitic stainless steel, plasma nitriding, corrosion resistance, potentiodynamic polarization, electrochemical impedance spectroscopy

## Abstract

The application possibilities of austenitic stainless steels in high friction, abrasion, and sliding wear conditions are limited by their inadequate hardness and tribological characteristics. In order to improve these properties, the thermochemical treatment of their surface by plasma nitriding is suitable. This article is focused on the corrosion resistance of conventionally plasma-nitrided AISI 304 stainless steel (530 °C, 24 h) in 0.05 M and 0.5 M sodium chloride solutions at room temperature (20 ± 3 °C), tested by potentiodynamic polarization and electrochemical impedance spectroscopy. Optical microscopy, scanning electron microscopy, energy-dispersive X-ray spectroscopy, and X-ray photoelectron spectroscopy are used for nitrided layer characterization. The experiment results confirmed the plasma-nitrided layer formation of increased micro-hardness related to the presence of Cr_2_N chromium nitrides and higher surface roughness compared to the as-received state. Both of the performed independent electrochemical corrosion tests point to a significant reduction in corrosion resistance after the performed plasma nitriding, even in a solution with a very low chloride concentration (0.05 mol/L).

## 1. Introduction

Austenitic stainless steels are commonly used in various fields of human activity because of the combination of high corrosion resistance and satisfactory mechanical properties. However, their utilization as a construction material in applications where they are subjected to friction, abrasion, or sliding wear is limited by their insufficient hardness and poor tribological properties [1,2,3,4,5,6]. The above problem can be solved by nitriding, which involves the diffusion of nitrogen into the material’s surface layer. The nitrided surface has increased hardness and can exhibit lower friction coefficients, reducing friction between moving parts. This contributes to improved efficiency and reduced energy consumption. Additionally, the nitrided layer can act as a solid lubricant in some cases [7,8,9]. Older methods of nitriding were economically demanding and caused distinctive environmental problems [10,11,12].

Plasma nitriding (PN) takes place in ionized gases (N₂ + H₂, NH_3_) at reduced pressure under the influence of an electric field. It is less time-consuming and is environmentally friendly because it typically uses fewer hazardous chemicals than previous nitriding methods and produces minimal waste [8,9,13,14,15]. By improving hardness and wear resistance, plasma nitriding helps extend the life of austenitic stainless-steel components, which is critical in industries in which durability and longevity are essential factors [11,12,13,14,15].

The PN of stainless steels has been the subject of research for several decades, and many specific methods based on it are currently in use. The choice of method depends on factors such as the material being treated, the desired outcome, and the characteristics of the components undergoing nitriding. The ways of PN differ, especially in key process parameters (temperature, gas composition, pressure, and treatment duration), which affect the resulting properties of the nitrided stainless-steel surfaces [16,17,18,19,20]. The obtained hardness is influenced mainly by the nitriding temperature used and depends on the precipitation of hard chromium nitrides, which are typically formed at temperatures above 500 °C. Therefore, this conventional high-temperature plasma nitriding brings a significant increase in hardness and a high resistance to wear, but due to the depletion of chromium, the corrosion resistance is limited [13,18,19,20,21,22,23,24,25,26]. After high-temperature PN, the loss of the passive behavior of austenitic stainless steels in potentiodynamic polarization has been observed by several authors [8,15,17,18]. In some cases, high-temperature nitriding can also lead to a martensitic transformation of the austenitic structure [27,28,29]. Because martensite is a harder phase than austenite, further enhancing of the material’s hardness occurs [29].

However, if the nitriding temperature is around 400 °C (so-called low-temperature nitriding), an expanded austenite (“S” phase) as an oversaturated solid solution of nitrogen in the austenitic face-centered cubic lattice is created [13,14,15,19,20,22,24,26]. This process is not accompanied by chromium depletion, and the material retains its high corrosion resistance [14,19,20,21,22]. A decrease in susceptibility to the pitting corrosion in chloride solutions after low-temperature nitriding has been confirmed by authors [23,24,25]. The expanded austenite contributes to increased hardness, but the effect is not as significant as the formation of hard chromium nitrides [9,13,17]. Moreover, a considerable amount of residual austenite may remain in the structure. The authors [17] noted that the micro-hardness of AISI 316L stainless steel after 4 h low-temperature nitriding at 400 °C (about 580 HV) is approximately half that observed in high-temperature nitriding at 520 °C (about 1200 HV). The choice between low- and high-temperature austenitic stainless-steel plasma nitriding depends on the specific application requirements. If, in addition to improved tribological properties, maintaining the austenitic structure and low susceptibility to corrosion are important, low-temperature nitriding may be preferred. For maximum hardness and wear resistance, high-temperature nitriding is more suitable. A compromise between low-temperature and high-temperature nitriding is rapid, short-term, high-temperature nitriding, which results in a nitrided layer that contains both expanded austenite and chromium nitrides [30,31].

The presented paper deals with the properties of plasma-nitrided AISI 304 stainless steel. The plasma nitriding process was performed in an industrial company producing stainless-steel components with an improved hardness and tribological properties for various applications, including those for which contact with variously concentrated chloride solutions is common. The main aim is to assess to what extent this conventional plasma nitriding (530 °C, 24 h) reduces the corrosion resistance of a given material in chloride solutions, dependent on the chloride concentration used. The plasma-nitrided layer is characterized by optical microscope (OM), SEM, EDX, and X-ray photoelectron spectroscopy (XPS). The micro-hardness of the PN layer and the surface roughness parameters are assessed as well. The evaluation of the corrosion resistance is based on potentiodynamic polarization (PP) and electrochemical impedance spectroscopy (EIS), both carried out in 0.05 M and 0.5 M sodium chloride solutions at room temperature (20 ± 3 °C).

## 2. Materials and Methods

The experimental material AISI 304 was purchased as a 1.5 mm thick sheet with a smooth and matt surface (2B surface finish) and the following chemical composition (wt.%) specified by atomic emission spectrometry (Spectromaxx analyzer, SPECTRO Analytical Instruments GmbH, Kleve, Germany): Cr 19.09, Ni 8.15, Mn 1.33, N 0.074, C 0.03, Si 0.37, P 0.029, S 0.004, Fe balance.

For the PN process, rectangular specimens with dimensions of 15 mm × 40 mm × 1.5 mm were prepared. A part of the specimens were left in their original state (“as-received state”) for a comparison of the obtained tests results. The surfaces of the specimens to be nitrided were not mechanically or chemically treated, only rinsed with water and ethanol and then dried with a stream of air.

The PN process was performed in the specialized laboratory of Rübig SK, k.s. (Prievidza, Slovakia) in a plasma nitriding furnace (the plasma nitriding process was identical to that used by this company for nitriding the manufactured components). The recipient (furnace casing) was connected to the circuit as an anode. The process lasted 24 h and was carried out at a temperature of 530 °C. The process included argon dusting. The nitriding was followed by cooling to a temperature of 160 °C, after which, the pressure was equilibrated to atmospheric pressure, and nitrided specimens were cooled freely to a common laboratory temperature.

For characterization, the nitrided specimen’s surface was displayed and EDX mapping analyzed by a Tescan Vega scanning electron microscope (SEM). The cross-section of the plasma-nitrided specimen was observed by optical microscope (OM) after glycerine + HNO_3_ + HF etching.

The presence of chromium nitrides and the prevalent type were determined by XPS analysis performed in a specialized laboratory at the A. Chełkowski Institute of Physics, University of Silesia (Katowice, Poland), using a Physical Electronic XPS spectrometer (Physical Electronics PHI 5700, Lake Drive East Chanhassen, Chanhassen, MN, USA). For photoelectron excitation, the X-ray Al_Kα1_ monochromatic radiation with energy of 1486 eV was used. The photoelectron survey and high-resolution spectra were collected from 80 μm diameter areas of the plasma-nitrided specimen, both before and after surface cleaning (Ar^+^, 1.5 kV, 20 mA, 5 min). The photoelectron spectra were analyzed using MULTIPAK (v.9.6.0.1, ULVAC PHI) software. The high-resolution spectra were calibrated using the C1s peak (284.6 eV).

The Vickers micro-hardness evaluation of the plasma-nitrided specimen was performed using a Zwick/Roell ZHVμ micro-hardness tester (Taicang, China) with a load of 0.01 kp (HV0.01 = 0.0981 N) and with a 10 s action of the indenter. For better visibility of the resulting indentations, the specimen was metallographically prepared (without etching) before the micro-hardness measurement. The micro-hardness measurement was performed in one measuring line.

The roughness parameter measurement was carried out by the Mitutoyo SJ 400 Rough-ness Tester (Kanagawa, Japan). For the as-received and plasma-nitrided surface, the roughness profile in the central part of the specimen (longitudinal direction, 5 sections of length 0.8 mm, i.e., a total of 4 mm) was extracted. The resulting values of the roughness parameters were obtained as the arithmetic mean.

Corrosion tests were carried out at the temperature 20 ± 3 °C in pH-neutral 0.05 M and 0.5 M sodium chloride solutions. The sodium chloride used was analytical grade.

The electrochemical corrosion tests (electrochemical impedance spectroscopy—EIS and potentiodynamic polarization—PP) were performed in the conventional three-electrode cell system with a saturated calomel electrode (SCE) and a platinum auxiliary electrode (Pt) using the BioLogic corrosion measuring system with a PGZ 100 measuring unit (Seyssinet-Pariset, France). The time for potential stabilization between the specimen and the electrolyte was set to 10 min. The exposed area of the specimen was 1 cm^2^. An overview of the specimen designations for the PP and EIS tests is given in Table 1.

Electrochemical impedance spectroscopy measurements were recorded at the corrosion potential over a frequency range from 100 KHz to 5 mHz (10 points per decade, 10 mV R.M.S amplitude). The results of the measurements were displayed as the Nyquist and Bode plots. The representative Nyquist curve (to display in the Figure) was selected from three measurements for the same type of surface conditions (AR/PN) and the same composition of solution (0.05 M/0.5 M). The EIS parameter values were obtained by EC-Lab software, V11.10 (Z-fit analysis of the Nyquist curves). The arithmetic means and standard deviations were calculated from three measurements under the same conditions (the same type of surface and composition of solution).

The potentiodynamic polarization curves were recorded at the sweep rate of 1 mV/s. A potential scan range was applied between −0.3 and 0.8 V vs. open circuit potential (OCP). At least three experiment repeats were carried out for each combination of conditions (AR/PN surface; 0.05 M/0.5 M solution), and the representative curve was selected (to display in the Figure). Arithmetic means and standard deviations were calculated in the above-mentioned manner.

## 3. Results and Discussion

### 3.1. Characterization of the Nitrided Layer

**Figure 1:**
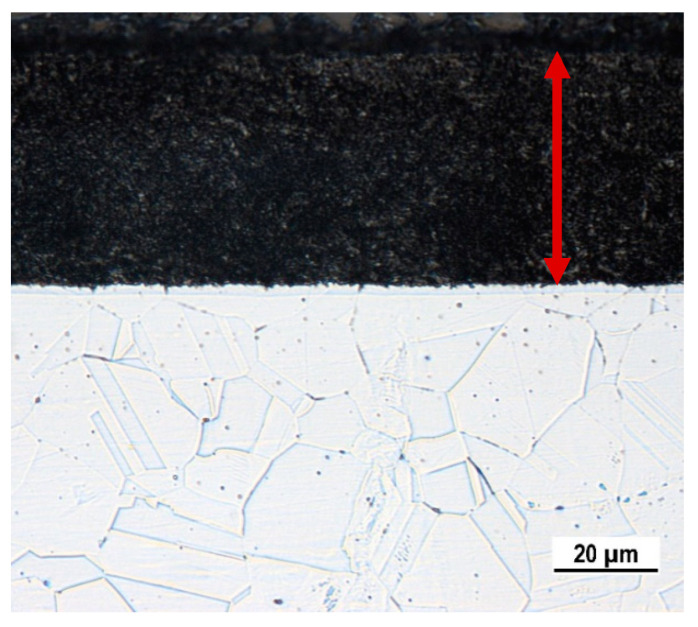
A cross-section edge of the plasma-nitrided specimen: OM, glycerine + nitric acid + hydrofluoric acid etching (the thickness of PN layer is marked by the red arrow).

**Figure 2:**
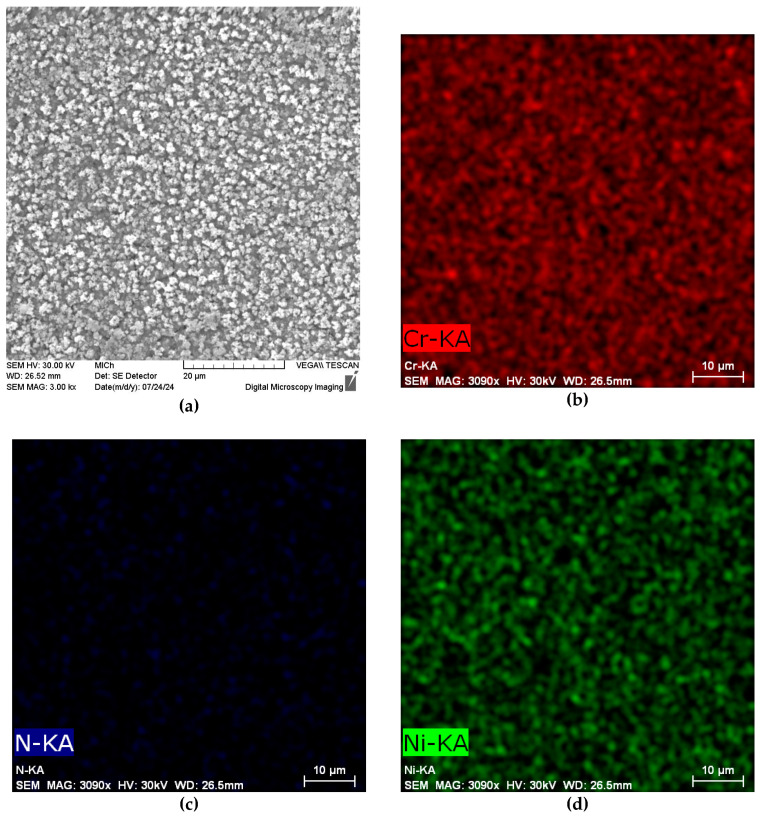
Plasma-nitrided surface of AISI 304 specimen: (**a**) morphology shown by SEM; (**b**) distribution of chromium; (**c**) distribution of nitrogen; and (**d**) distribution of nickel, shown by EDX analysis.

**Figure 3:**
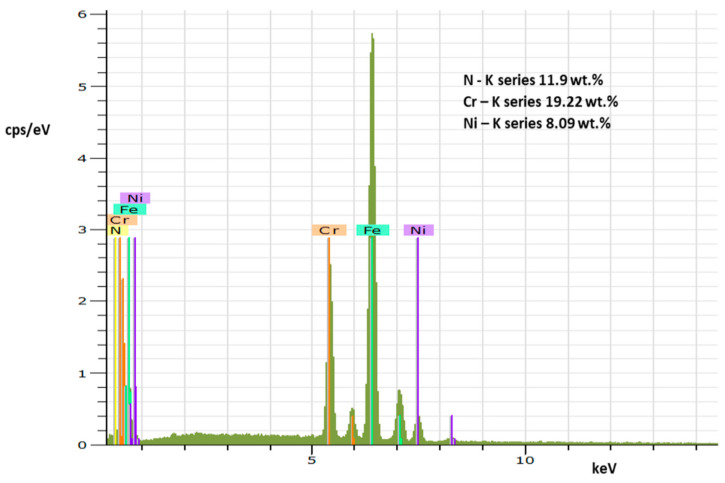
The EDX spectrum of the selected chemical elements.

As the result of the applied PN process, a nitrided surface layer (approx. 45 µm thickness) observable by OM in the specimen cross-section was formed (Figure 1). The morphology of the nitrided surface obtained by SEM analysis is presented in Figure 2a. The distribution of chromium, nitrogen, and nickel displayed by EDX mapping is shown in the Figure 2b–d. The corresponding EDX spectrum of these chemical elements is presented in Figure 3. As can be seen, the resulting surface is discontinuous and rough and exhibits an uneven distribution of nitrogen (Figure 2b).

The increased roughness of the PN surface compared to the as-received one was reflected in the values of the roughness parameters: arithmetical mean deviation of the assessed profile (R_a_), the average maximum peak-to-valley height (R_z_), and the profile slope (R_sk_), listed in Table 2. In general, the surface roughness of stainless steel significantly affects the susceptibility to corrosion. It disrupts the uniformity and stability of the passive layer and introduces defects into the microstructure. The increased surface area exposed to the corrosion environment enhances the adsorption of chlorides and conditions the presence of electrochemical heterogeneities [32,33]. All the mentioned aspects can play an important role also in the case of the rough plasma-nitrided stainless steel surface.

**Figure 4:**
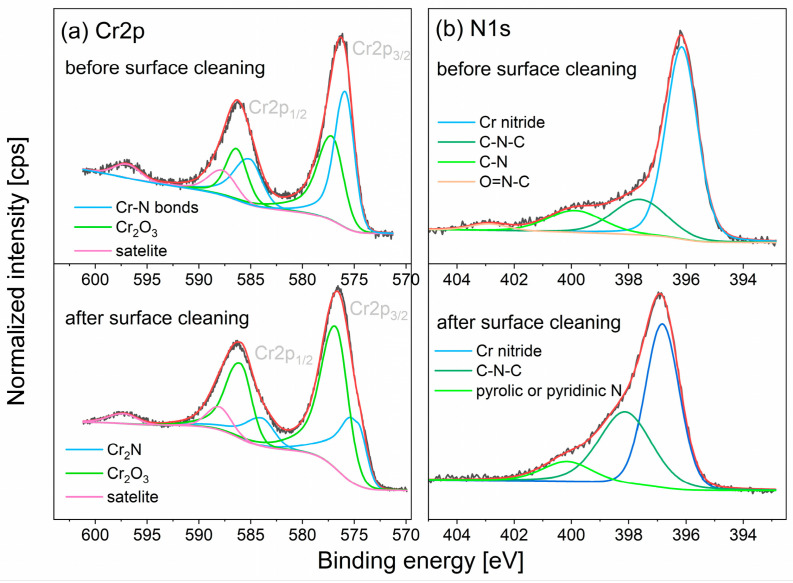
High-resolution spectra of chromium and nitrogen: (**a**) Cr2p and (**b**) N1s on plasma-nitrided layer before and after Ar^+^ cleaning.

The performed XPS analysis aimed to identify the chemical states of the chromium (Figure 4a) and nitrogen (Figure 4b) of the surface of the plasma-nitrided specimen. If not indicated otherwise, the identification of their chemical states was carried out using the NIST X-ray Photoelectron Spectroscopy Database [34]. Chromium exists in two chemical states; before surface cleaning, the Cr2p_3/2_ peak at a binding energy of 575.87 eV indicates the presence of chromium in a nitrogen environment [35], while the peak at an energy of 577.17 eV can be assigned to Cr_2_O_3_ [35,36] or chromium(III) hydroxide Cr(OH)_3_ [36]. In addition, chromium satellite lines appeared at energies of 587.71 eV and 596.91 eV. After surface cleaning, slight shifts in the main peaks were observed; the Cr2p_3/2_ peak corresponding to Cr_2_N appeared at a binding energy of 574.73 eV [37], and the peak corresponding to Cr_2_O_3_ at 576.77 eV [38]. The N1s line revealed the nitrogen present in multiple chemical states; the predominant contribution to the N1s line corresponds to chromium nitrides. The N1s peak at 396.13 eV observed before surface cleaning can be identified as oxynitride, whereas the sputtered surface peak at 396.83 eV can be assigned to Cr_2_N [39]. The Cr_2_N phase was also confirmed by the authors [15,17] after high-temperature nitriding of austenitic stainless steels.

The nitriding temperature used obviously ensured sufficient solubility and the appropriate kinetic conditions for the intensive diffusion of nitrogen atoms into the surface layer of the material and their reactions with chromium. Due to the sufficient thermal energy of chromium atoms, the formation of chromium nitride was thermodynamically favored, unlike the lower temperatures, at which the driving force for the formation of stable nitride phases is insufficient [40,41].

### 3.2. Micro-Hardness Measurement

**Figure 5:**
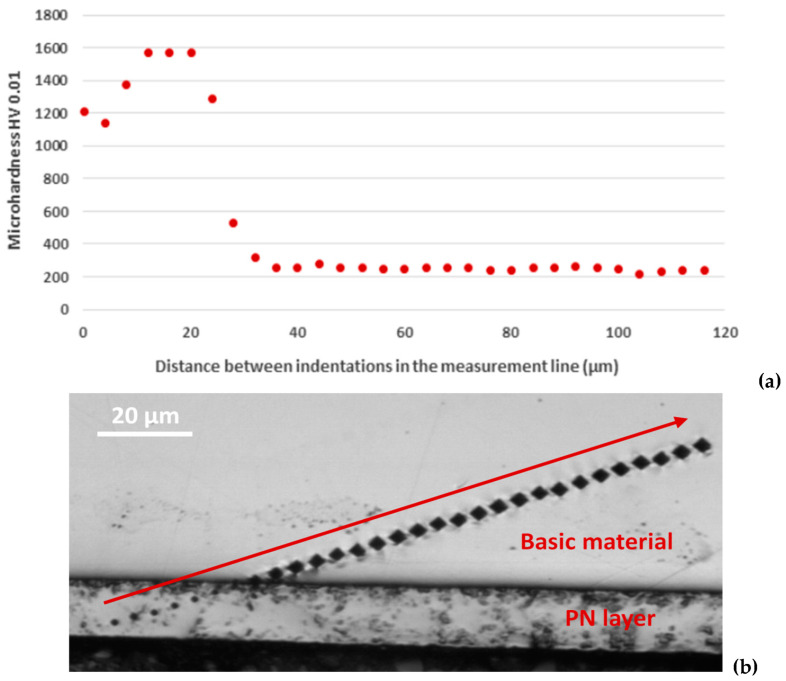
Course of AISI 304 micro-hardness after plasma nitriding: (**a**) graph of micro-hardness, (**b**) measurement line.

The sequence of micro-hardness measurements after PN and the obtained values are documented in Figure 5. The micro-hardness of the nitrided layer ranged from 1143 to 1572 HV 0.01, followed by a steep transition to the softer part of the specimen with micro-hardness values between 223 and 286 HV 0.01. The high hardness of the material after PN at temperatures above 500 °C is related to the Cr_2_N chromium nitride, which was formed under these conditions (Section 3.1) and significantly increased the hardness of the material [9,16,17,42,43]. The comparable values of the PN layer micro-hardness (about 1300 HV) on austenitic stainless steel plasma nitrided at 520 °C were recorded by de Araújo et al. [17].

### 3.3. Potentiodynamic Polarization (PP)

**Figure 6:**
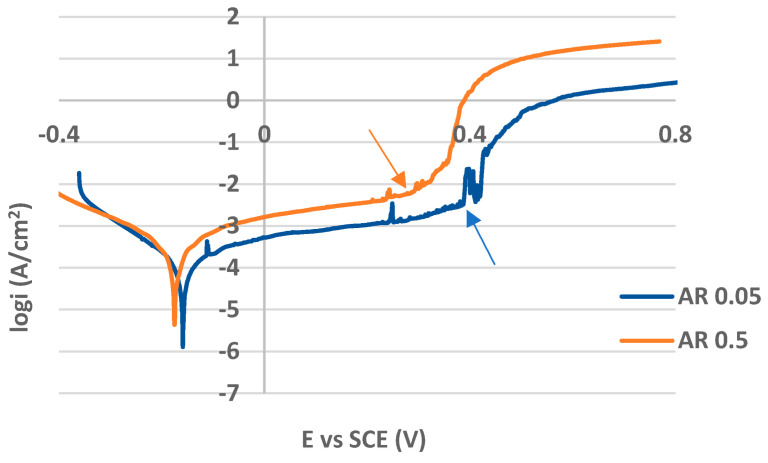
The potentiodynamic polarization curves for the as-received AISI 304 in 0.05 M and 0.5 M sodium chloride solutions (E_p_ locations are marked by the arrows).

**Figure 7:**
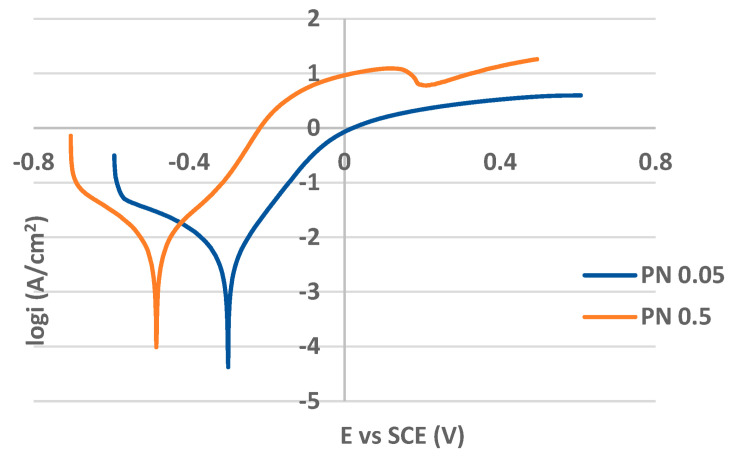
Potentiodynamic polarization curves for plasma-nitrided AISI 304 in 0.05 M and 0.5 M sodium chloride solutions.

The PP curves for the as-received (non-treated) and plasma-nitrided surfaces in both 0.05 M and 0.5 M NaCl solutions are shown in Figure 6 and Figure 7, respectively. The values of PP parameters are listed in Table 3. While the curves in Figure 6 are typical for passivating metals, the curves for plasma-nitrided surfaces are without passive anodic branches, and they express the state of the active corrosion (Figure 7). This essential difference in the curve shapes points to the different control of the anodic dissolution rate. The dissolution rate for passivating metals is controlled by the passive current density and that for actively corroding metal by corrosion current density. Therefore, AR 0.05 and AR 0.5 curves were evaluated by the corrosion potential E_corr_ and the pitting potential E_p_ (Table 3). E_corr_ was identified directly from the curve as the potential of the transition from the cathodic to the anodic branch. E_p_, denoting the breakdown of the passive film and the start of the stable pit growth, was determined as the potential of the strong continual increase in the current density in the passivity region (E_p_ locations in Figure 6 are marked by the arrows). The higher E_p_ value points to the higher resistance to the pitting. The PP parameters E_corr_, i_corr_ (corrosion current density), β_a_, β_c_ (anodic and cathodic Tafel slopes), and v_corr_ (corrosion rate), for PN 0.05 and PN 0.5 curves (Figure 7), were obtained as the result of the Tafel extrapolation using EC-LAB software.

According to the PP curves and the E_p_ values, the as-received surfaces reflected adequate resistance to the pitting corrosion in both variously concentrated chloride solutions. Consistent with the published results [44,45,46,47,48], the higher chloride concentration induced the more intensive penetration of chloride anions through the weakened localities of the passive film, and it led to an E_p_ drop compared to the AR 0.05 E_p_ value (Table 3). The slight difference dependent on the chloride concentration was also recorded in the thermodynamic stability expressed by the E_corr_ values of the AR 0.05 and AR 0.5 specimens.

As shown in Figure 7, the applied plasma nitriding process caused the passivity loss of PN specimens even at a very low sodium chloride concentration (0.05 M solution). The kinetics of the corrosion process expressed by the corrosion current density and corrosion rate points to an active corrosion in both solutions and a decrease in the E_corr_ values substantiates lower thermodynamic stability compared to the as-received state (Table 3). In order to determine the rate-determining reaction of this corrosion process, the Tafel slope values (obtained as the results of the Tafel extrapolation using EC-LAB software) were used. Taking into account that the rate-determining step is usually associated with the reaction that has the less efficient kinetics (it means a higher Tafel slope), in both the solutions, the corrosion mechanism seems to be controlled primarily by the kinetics of the cathodic reaction [49,50].

Generally, the values of the PP parameters (Table 3) indicate a reduced corrosion resistance in both solutions compared to the as-received state. This result could be related to the precipitation of chromium nitride that depletes the surrounding areas of chromium, reducing the amount of the chromium atoms available to form the protective passive oxide film [17,18].

**Figure 8:**
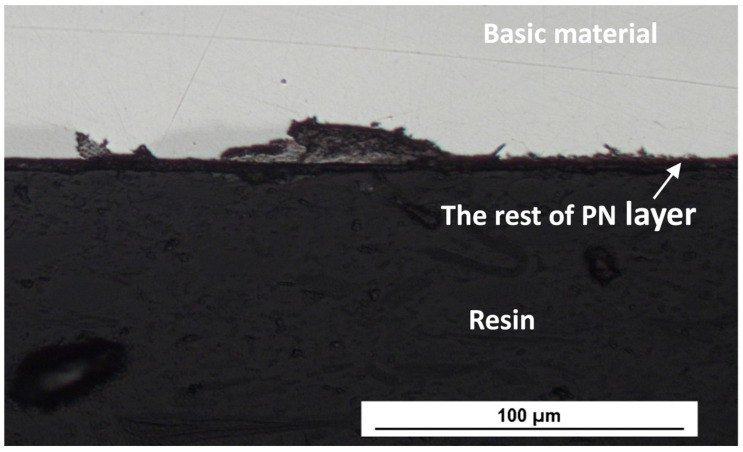
The corrosion damage created during the potentiodynamic polarization test in a 0.05 M NaCl solution; the edge of the cross-section of the specimen.

In addition, the presented rough morphology of the plasma-nitrided surface (Figure 2a), with an uneven distribution of nitrogen (Figure 2c), and the increased roughness parameters point to the presence of discrete, separated chromium–nitrogen precipitates, which do not form a continuous layer and enable the intensive scattered penetration of chloride anions into the material. The heterogeneity introduced by the rough surface and by discrete Cr_2_N precipitates obviously conditioned a formation of the sites of the active corrosion (oxidation of iron: Fe → Fe^2+^ + 2e^−^) and the cathodic sites (reduction of oxygen in the aqueous solution: O_2_ + 2H_2_O + 4e^−^ → 4OH^−^). Consequently, the hydrolysis of Fe^2+^ cations in the presence of Cl^-^ anions proceeded (Fe^2+^ + 2H_2_O + 2Cl^−^ → Fe(OH)_2_ + 2H^+^ + 2Cl^−^), and it led to an acidification of the environment and to a further acceleration of the corrosion reactions. The result was active corrosion during potentiodynamic polarization, indicating a deterioration in the corrosion resistance of the plasma-nitrided surface, which was not prevented even by a low chloride concentration (0.05 mol/L). Corrosion damage, which occurred during the potentiodynamic polarization test in the 0.05 M NaCl solution, is shown on the edge of the cross-section of the specimen for illustration (Figure 8).

De Araujo et al. [17] evaluated the corrosion behavior of AISI 316L stainless steel after ionic plasma nitriding performed at 520 °C for 4 h by potentiodynamic polarization in a 0.6 M NaCl solution. In agreement with our results, they also recorded the loss of passivity and almost the same shape of the curve. The authors Kartikasari et al. [18] came to similar results for the same stainless steel plasma nitrided at 500 and 550 °C for 3 h and tested by potentiodynamic polarization in an unspecified chloride solution. Contrary to the findings above, Mareci et al. [51] recorded a strengthening of the corrosion resistance from the potentiodynamic polarization of plasma-nitrided austenitic stainless steel (500 °C, 14 h) in 0.5 M NaCl. The obtained results were attributed to a continuous, smooth CrN layer prepared in a glow discharge plasma nitriding unit [51].

### 3.4. Electrochemical Impedance Spectroscopy (EIS)

**Figure 9:**
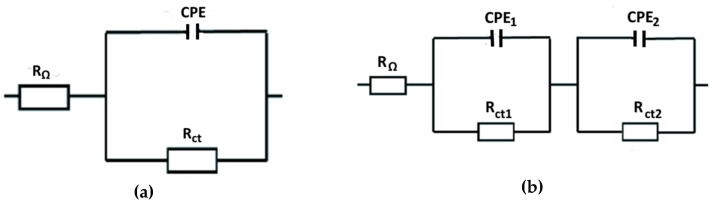
Equivalent circuits for the fitting of the impedance data: (**a**) circuit with one time constant, (**b**) circuit with two time constants.

Similar to the authors [51,52,53], the evaluation of the measured impedance spectra of the as-received specimens was based on the one time constant observed. Therefore, a single loop circuit consisting of electrolyte resistance (R_Ω_), charge transfer resistance (R_ct_), and the constant phase element (CPE) was applied (Figure 9a). For the plasma-nitrided specimens, the two-layer model of a porous surface film with two time constants containing R_Ω,_ R_ct1_, R_ct2_, CPE_1_, and CPE_2_ was used (Figure 9b) [51]. CPE was used instead of an ideal capacitor because, due to factors like surface roughness and heterogeneity, the tested systems (as-received surface/solution, PN surface/solution) exhibited non-ideal capacitive behavior [54]. CPE represents the capacitor if the “n” exponent present in the equation for the mathematical CPE expression equals one [55,56]. According to Kerner et al. [57], the capacitance dispersion on solid electrodes is related to the surface disorder (i.e., heterogeneities) and to the surface roughness (i.e., geometric irregularities) much more than to the atomic scale.

**Figure 10:**
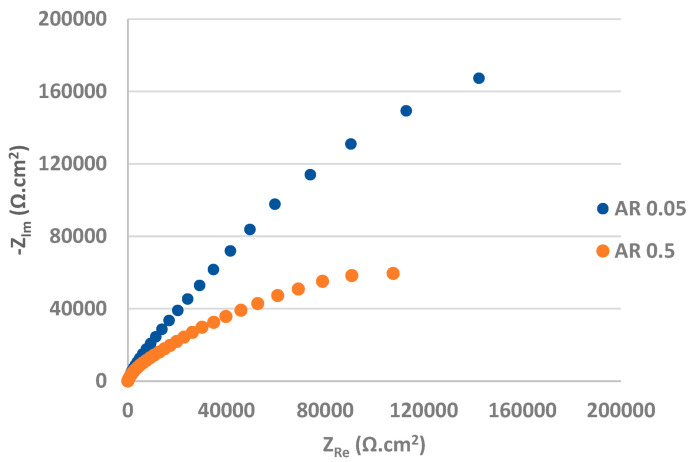
Nyquist curves for as-received AISI 304 in 0.05 M and 0.5 M sodium chloride solutions.

**Figure 11:**
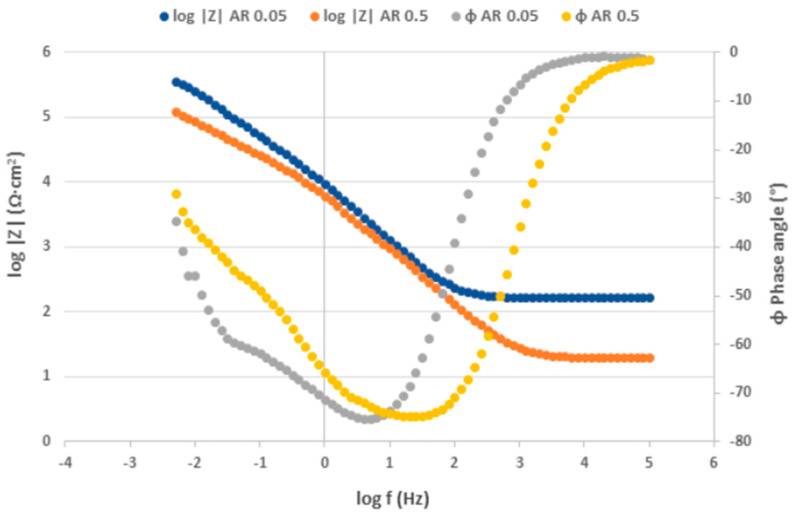
Bode curves for as-received AISI 304 in 0.05 M and 0.5 M sodium chloride solutions.

**Figure 12:**
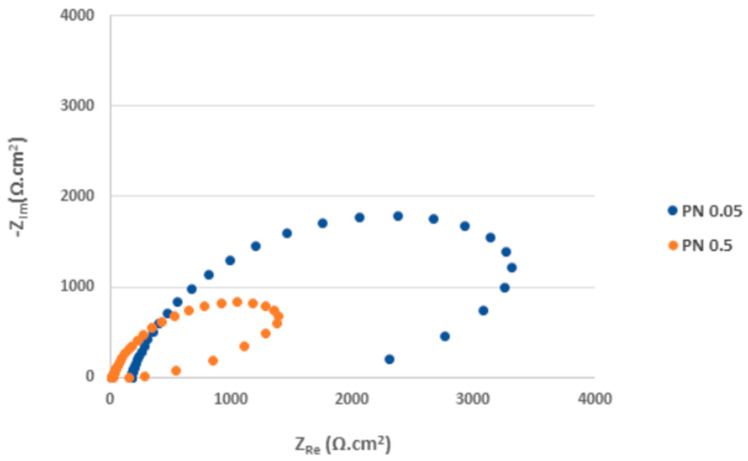
Nyquist curves for plasma-nitrided AISI 304 in 0.05 M and 0.5 M sodium chloride solutions. (The tail in the PN 0.5 Nyquist curve indicates that the impedance in low frequencies might be affected by transport processes, e.g., diffusion [60]).

**Figure 13:**
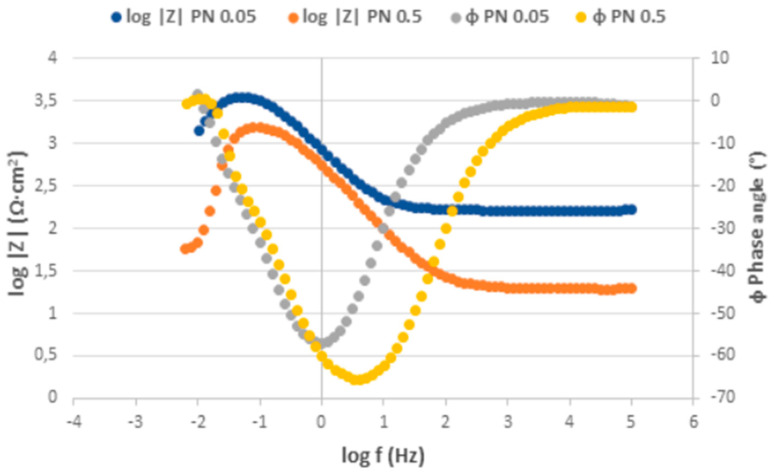
Bode curves for plasma-nitrided AISI 304 in 0.05 M and 0.5 M sodium chloride solutions.

The Nyquist plots obtained from the EIS measurement for as-received surface are displayed in Figure 10, the Bode plots expressing the dependence of impedance modulus |Z| and phase angle ɸ on the measured frequency are shown in Figure 11. The Nyquist and Bode plots for PN surface are shown in Figure 12 and Figure 13 respectively. The values of the fitted impedance parameters obtained by Z-fit extrapolation of the Nyquist curves (EC-LAB software) are presented in Table 4 and Table 5. The values of charge transfer resistance R_ct_ for the as-received surfaces, derived from the Nyquist curves (Figure 10), confirmed the presence of the passive surface film recorded also at the PP measurement [58]. This result is consistent with the Bode plots (Figure 11), showing the high values of impedance modulus |Z| in the low-frequency region, which points to the high corrosion resistance of the bare metal [59].

In contrast to the PP measurement, the effect of the chloride concentration on the quality and resistance of the passive oxide film was more pronounced during the EIS measurement (almost 6x lower R_ct_ in the 0.5 M NaCl solution compared to the 0.05 M solution). A significant decrease in the R_ct_ of the AISI 304 stainless steel with a rise in the concentration of the NaCl solution was also noted by Yin [48].

According to the EIS results (Figure 12, Table 5), the total R_ct_ values (R_ct1_ + R_ct2_), i.e., 4.48 kΩ·cm^2^ for PN 0.05 and 3.71 kΩ·cm^2^ for PN 0.5 specimens, are very low and less dependent on the chloride concentration compared to the AR ones. It points to the increased electrical conductivity of the plasma-nitrided surface leading to an acceleration of ongoing electrochemical reactions [56]. This result was confirmed by the Bode curves (Figure 13), in which significantly lower |Z| values compared to the as-received surface in the low-frequency region were recorded [59].

A higher electrical conductivity can be related to the discontinuous plasma nitride surface (Figure 2a) with more active sites for the adsorption and penetration of chloride anions and, consequently, for efficient charge transfer processes. Moreover, the precipitation of chromium nitrides causes chromium depletion in adjacent areas, supports the suppression of passive oxide film, and facilitates easier electron transfer [15,18,40].

## 4. Conclusions

- The plasma nitriding of AISI 304 stainless steel performed at a temperature of 530 °C for 24 h induced the formation of a nitrided surface layer (thickness approx. 45 µm) with a rough, discontinuous surface (Table 2) and with an uneven distribution of nitrogen.
- XPS analysis of the surface nitrided layer proved the presence of bonds between chromium and nitrogen. The predominant contribution to the N1s line corresponds to chromium nitrides; sputtered surface peak at 396.83 eV can be assigned to the Cr_2_N phase. This phase was also confirmed by the Cr2p_3/2_ peak that appeared at a binding energy of 574.73 eV.
- The plasma nitriding process significantly increased the micro-hardness of the surface layer compared to the inner parts of the material (1143 to 1572 HV 0.01 in the nitride layer; 223 to 286 HV 0.01 in the inner part).
- Potentiodynamic polarization revealed the loss of the passive behavior of the material after plasma nitriding in both solutions—the shape of PP curves and the i_corr_ values obtained by Tafel analysis (Table 3) are typical for an actively corroding metal. According to the Tafel slope values, the cathodic reaction was the rate-determining reaction in both of the NaCl solutions.
- The results of the EIS measurements show a significant decrease in total R_ct_ values after plasma nitriding (Table 4 and Table 5), which means an increase in the rate of electrochemical reactions at the material/solution interface.

It can be stated that the results of the EIS and potentiodynamic polarization measurements are in good agreement, and they confirmed a significant reduction in corrosion resistance after plasma nitriding (530 °C/24 h), even in a solution with a very low concentration of chlorides (0.05 mol/L). Therefore, it is not recommended to use AISI 304 steel thermochemically treated in the above-described way in any chloride solutions.

## Figures and Tables

**Table 1:** An overview of the specimen designations.

Specimen Designation	Type of Surface/Solution
AR 0.05	As-received, non-treated/0.05 M NaCl
AR 0.5	As-received, non-treated/0.5 M NaCl
PN 0.05	Plasma-nitrided/0.05 M NaCl
PN 0.5	Plasma-nitrided/0.5 M NaCl

**Table 2:** The values of roughness parameters: the arithmetical mean deviation of the assessed profile (R_a_), the average maximum peak-to-valley height (R_z_), and the profile slope (R_sk_).

Specimen Designation	R_a_ (μm)	Rz (μm)	R_sk_ (-)
AR	0.10	1.02	−1.58
PN	0.24	2.59	−1.63

**Table 3:** The values of the potentiodynamic polarization parameters.

Specimen Designation	Corrosion Potential E_corr_ (V vs SCE)	Pitting Potential E_p_ (V vs SCE)	Corrosion Current Density i_corr_ (10^−3^ mA/cm^2^)	Cathodic Tafel Slope β_c_ (V/Decade)	Anodic Tafel Slope β_a_ (V/Decade)	Corrosion Rate v_corr_ (mm/Year)
AR 0.05	−0.16 ± 0.02	0.39 ± 0.04	-	-	-	-
AR 0.5	−0.18 ± 0.03	0.29 ± 0.05	-	-	-	-
PN 0.05	−0.30 ± 0.03	-	3.19 ± 0.19	0.15 ± 0.05	0.10 ± 0.05	0.04 ± 0.002
PN 0.5	−0.48 ± 0.05	-	6.81 ± 0.21	0.18 ± 0.04	0.15 ± 0.05	0.08 ± 0.002

**Table 4:** The values of the fitted impedance parameters for the as-received specimens: R_Ω_—electrolyte resistance, R_ct—_charge transfer resistance, CPE—constant phase element, n—exponent.

Specimen Designation	R_Ω_ (kΩ·cm^2^)	R_ct_ (kΩ·cm^2^)	n	CPE (µF/cm^2^)
AR 0.05	0.161 ± 0.004	236.68 ± 0.9	0.85 ± 0.003	24.64 ± 0.12
AR 0.5	0.019 ± 0.002	40.68 ± 0.2	0.85 ± 0.002	33.5 ± 0.18

**Table 5:** The values of the fitted impedance parameters for plasma-nitrided specimens: R_Ω_—electrolyte resistance; R_ct1_, R_ct2_—charge transfer resistances; CPE_1_, CPE_2_—constant phase elements; n_1_, n_2_—exponents.

Specimen Designation	R_Ω_ (kΩ·cm^2^)	R_ct1_ (kΩ·cm^2^)	R_ct2_ (kΩ·cm^2^)	CPE_1_ (µF/cm^2^)	CPE_2_ (µF/cm^2^)	n_1_	n_2_
PN 0.05	0.122 ± 0.003	2.11 ± 0.1	3.37 ± 0.2	247 ± 1.2	14.82 ± 0.8	0.87 ± 0.002	0.50 ± 0.002
PN 0.5	0.018 ± 0.002	1.95 ± 0.1	1.76 ± 0.2	376 ± 1.4	6.11 ± 0.2	0.82 ± 0.002	0.75 ± 0.002

## Data Availability

Data are contained within the article.
